# Cardiogenic Shock in a Hemodialyzed Patient on Flecainide: Treatment with Intravenous Fat Emulsion, Extracorporeal Cardiac Life Support, and CytoSorb® Hemoadsorption

**DOI:** 10.1155/2019/1905871

**Published:** 2019-07-24

**Authors:** Nicolas De Schryver, Philippe Hantson, Vincent Haufroid, Mélanie Dechamps

**Affiliations:** ^1^Department of Intensive Care, Clinique St-Pierre, 1340 Ottignies, Belgium; ^2^Department of Intensive Care, Cliniques St-Luc, Université catholique de Louvain, 1200 Brussels, Belgium; ^3^Louvain Centre of Toxicology and Applied Pharmacology, Université catholique de Louvain, 1200 Brussels, Belgium; ^4^Department of Clinical Chemistry, Cliniques St-Luc, Université catholique de Louvain, 1200 Brussels, Belgium; ^5^Cardiovascular Intensive Care, Cliniques St-Luc, Université catholique de Louvain, 1200 Brussels, Belgium

## Abstract

A 67-year-old woman with a history of end-stage renal disease on hemodialysis received a therapeutic dose (150 mg daily) of flecainide for three weeks. She was admitted to the Emergency Department for malaise and dizziness, and the electrocardiogram revealed ventricular tachycardia treated by amiodarone. Hemodynamic condition remained stable, and the toxicity of flecainide was initially not suspected until she developed within 8 hours a cardiogenic shock requiring vasopressors. The patient then received sodium bicarbonate (300 mmol) and dobutamine but experienced cardiac arrest two hours later. The administration of intravenous fat emulsion (IFE) was associated with return of spontaneous circulation, but there was a relapse of cardiovascular shock at the end of IFE infusion. The patient was placed on extracorporeal cardiac life support (ECLS), continuous hemofiltration, and hemoadsorption using the CytoSorb® cartridge. Serial determinations of serum flecainide concentration were obtained during the course of hemoadsorption, with a terminal half-life of 3.7 h during the first four hours and a global plasma clearance of 40.3 ml/min over the first 22 hours. The weaning of ECLS was possible on day 7. Intravenous fat emulsion infusion was followed by a significant increase in serum flecainide concentration. In addition, while conventional techniques of extrarenal epuration usually appear as poorly effective for flecainide removal, a mean plasma clearance of 40.3 ml/min was observed using the hemoadsorption technique based on CytoSorb® cartridge. However, the impact on the clinical course was probably extremely modest in comparison with ECLS.

## 1. Introduction

Flecainide is a Vaughan Williams class 1C antiarrhythmic drug influencing the fast inward sodium channel during the phase 0 of the action potential. Overdoses with flecainide have been associated with a high mortality rate (22.5%) in comparison with other class 1C molecules. Toxicity is less easily recognized in case of chronic therapy. Ventricular arrhythmias and decreased cardiac contractility in relationship with conduction disturbances are the main complications of flecainide overdose [1]. When the usual cardiopulmonary resuscitation with sodium bicarbonate and catecholamines is failing, rescue therapy with extracorporeal cardiac life support (ECLS) should be promptly considered. We report a case of flecainide toxicity following its therapeutic use in a patient with end-stage renal disease (ESRD). In addition to ECLS, the patient was also treated with intravenous fat emulsion (IFE) and an extrarenal epuration technique based on hemoadsorption (CytoSorb®).

## 2. Case Presentation

A 67-year-old woman (46 kg weight) underwent an aortic valve repair with aortic root replacement for severe dilation of the ascending aorta. In the postoperative period, she developed atrial fibrillation and was then prescribed flecainide two days before hospital discharge. Her other relevant medical history included ESRD requiring chronic hemodialysis, hypertension, and hypothyroidism. Her daily treatment included flecainide 150 mg od, losartan 100 mg od, acenocoumarol, levothyroxine 75 *μ*g od, pantoprazole 40 mg od, calcitriol 0.25 mg od, and cinacalcet 30 mg od. Three weeks after hospital discharge, the patient suddenly felt dizzy and was referred at night to the Emergency Department. A 12 lead electrocardiogram showed an extremely broad QRS complex tachycardia at a rate of 115 bpm and without P waves. The negative concordance of the QRS complexes in the precordial leads, as well as the extreme right QRS axis deviation, was consistent with the diagnosis of ventricular tachycardia. The i.v. administration of 300 mg amiodarone resulted in a decrease of heart rate to 78 bpm with a blood pressure of 122/76 mmHg. A second ECG showed a regular rhythm without P waves and with broad QRS complexes (189 ms) and with diffuse repolarization abnormalities. Relevant laboratory investigations were arterial pH 7.44, bicarbonate 22 mmol/l, lactate 1.9 mmol/l, serum creatinine 9.57 mg/dl, urea 99 mg/dl, and hsTnI 40 ng/l. Liver function tests were normal. Blood was also drawn for toxicological investigations, but the results were not immediately available. The patient was admitted in the Intensive Care Unit (ICU) for further monitoring of blood pressure and heart rhythm. Systolic blood pressure remained initially above 100 mmHg, heart rate around 75/min, and arterial pH above 7.45. Eight hours after hospital admission, blood pressure progressively decreased and norepinephrine was started. The lactate level started to increase at the same time (7.7 mmol/l). The ICU physician on duty reconsidered the initial diagnosis of ventricular tachycardia as a sign of flecainide toxicity. Transthoracic echocardiography showed a marked interventricular dyssynchrony with severely decreased systolic function of both ventricles. Left ventricular ejection fraction was calculated using the biplane Simpson method at 36%. Cardiac output was calculated at 1920 ml/min. Administration of sodium bicarbonate (300 mmol), calcium gluconate (4 g), and dobutamine was initiated. Over the next 3 hours, the norepinephrine requirements rapidly increased up to 1.5 mcg/kg/min. Lactate levels increased up to 10 mmol/l, and neurological status worsened. The patient was intubated, and mechanical ventilation was started but cardiac arrest (pulseless electrical activity rapidly followed by asystole) occurred. Cardiopulmonary resuscitation (CPR) was immediately performed with 1 mg epinephrine administration every 3 minutes. CPR was briefly interrupted every 2 minutes for rhythm analysis but asystole persisted. Thirteen minutes after the initiation of CPR, intravenous fat emulsion (20% Intralipid®) (IFE) bolus (1.5 ml/kg) was given over 1 minute and was followed one minute later by return of spontaneous circulation. Continuous infusion of IFE was continued at a rate of 0.25 ml/kg/min immediately after bolus administration for a total of 450 ml administered. At the same time, hemodynamics dramatically improved with complete weaning of norepinephrine requirements but without perceptible shortening of the QRS complexes. Five minutes after the end of IFE administration, blood pressure again dropped and norepinephrine was restarted with increasing doses up to 1.5 mcg/kg/min. Initiation of an extracorporeal cardiac life support (ECLS) and transfer to a tertiary center were then decided. In the meantime, profound hypotension occurred (46/28 mmHg). A second bolus of IFE (1.5 ml/kg) was administered, followed by continuous infusion at a rate of 0.25 ml/kg/min. At the time of venoarterial cannulation for ECLS two hours later by the mobile team of the tertiary hospital, norepinephrine and epinephrine requirements peaked at 2.6 mcg/kg/min and 0.5 mcg/kg/min, respectively. The patient had received an additional dose of 200 mmol of sodium bicarbonate. The lactate level was 19.9 mmol/l. IFE administration was discontinued immediately after cannulation. A total volume of 20% IFE administration was 1500 ml (33 ml/body weight kg).

**Figure 1:**
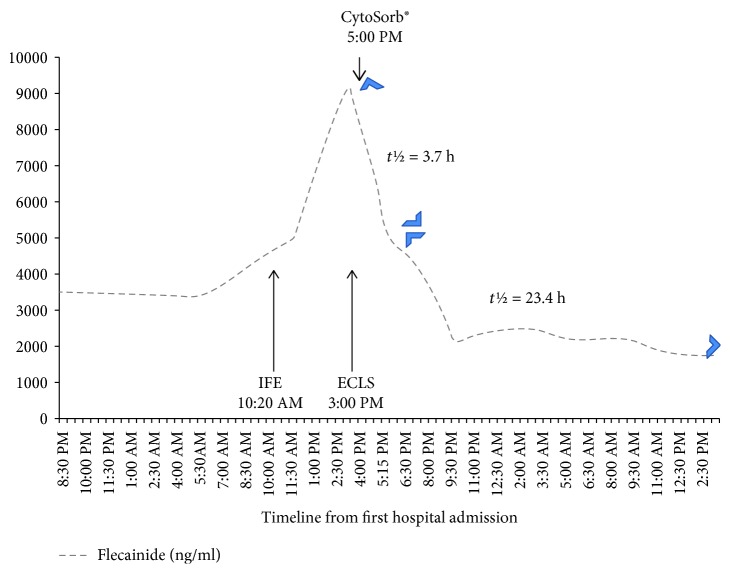
Evolution of serum flecainide concentration over time. IFE: intravenous fat emulsion. The terminal half-life (*t*½) elimination of flecainide was calculated over the first four hours (3.7 hours) following the introduction of CytoSorb® hemoadsorption and over the eighteen following hours (23.4 hours). Note that the terminal half-life was influenced by the combination of CytoSorb®, CVVHDF, and ECLS clearances and of metabolic (hepatic) clearance.

**Figure 2:**
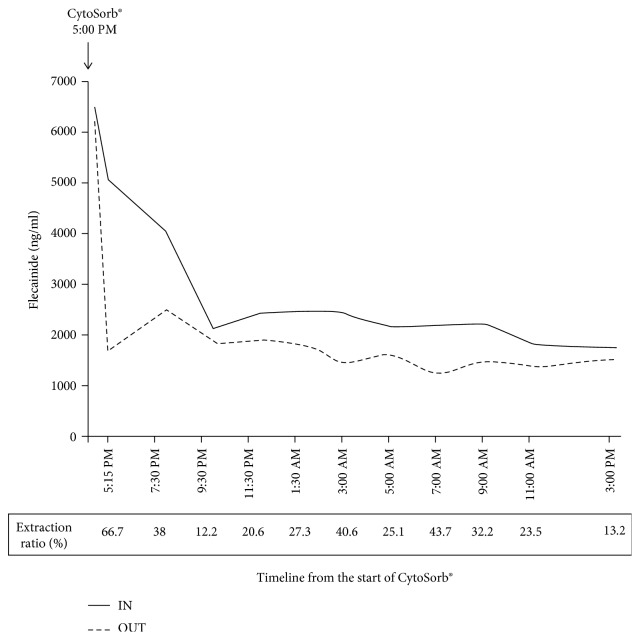
Determination of the serum flecainide concentration at the inlet (IN) and outlet (OUT) of the CytoSorb® hemoadsorption circuit. The extraction ratio (%) could be expressed as (concentration IN‐concentration OUT/concentration IN). Blood flow in the circuit was 200 ml/min. Plasma flow rate was expressed as blood flow rate × (1‐haematocrit).

After transfer to the tertiary center, continuous venovenous hemodiafiltration (CVVHDF) and additional hemoadsorption using CytoSorb® cartridge were started with a blood flow of 200 milliliters per minute. The detailed toxicokinetics of flecainide in relationship with the epuration technique are presented in Figures 1 and 2. The serum flecainide concentration was 3477 ng/ml at the time of admission in the first hospital, well above the therapeutic range (200-1000).

Within 72 hours, norepinephrine requirement dropped to less than 0.2 *μ*g/kg/min and QRS duration on ECG was reduced to 98 msec (atrial flutter). After 7 days, echocardiography showed a normal biventricular systolic function and ECLS could be weaned and removed. The patient maintained normal liver function tests over the ICU stay. Further clinical course was marked by pulmonary and abdominal infections, slow respiratory weaning, and gastrointestinal bleeding. The patient could be discharged to the ward on day 34, with an excellent neurological outcome and recovery of left ventricular function, and had a 6-month uneventful follow-up.

Genetic testing for CYP2D6 polymorphism was obtained. The investigated alleles were CYP2D6 ^∗^1, ^∗^2, ^∗^3, ^∗^4, ^∗^5, ^∗^6, ^∗^7, ^∗^8, ^∗^9, ^∗^10, ^∗^11, ^∗^15, ^∗^17, ^∗^29, ^∗^35, ^∗^41, duplication, and deletion. The patient genotype was confirmed to be CYP2D6^∗^1/^∗^4.

## 3. Discussion

Flecainide has similar characteristics to other medications for which IFE therapy has been successfully utilized. Flecainide has a large volume of distribution (*V*_D_ (4.9 L/kg)) and a high Log P (octanol/water) coefficient (3.8). This would predict a favorable response to IFE therapy in case of overdose with refractory shock [2]. The hypothesis of a “lipid sink” resulting in a sequestration of lipophilic medications from the receptors sites seems to be supported by some clinical observations contrasting with experimental data. Indeed, in a rabbit experimental model of flecainide poisoning, ILE administration did not result in an increase of plasma flecainide concentration [3]. Besides, IFE was not more effective than hypertonic sodium bicarbonate on the correction of QRS widening [3]. In a case of 2.5 g flecainide overdose, the intravenous infusion of a total of 1 L of 20% IFE was followed by an increase of serum flecainide concentration from 1800 ng/ml 100 minutes pre-IFE administration to 2760 ng/ml 7 hours post-IFE administration. In another patient admitted 90 minutes after a mixed overdose with 25 mg bisoprolol, 900 mg flecainide, and 225 mg aspirin, IFE therapy was administered soon after admission after that the patient had developed ventricular fibrillation. The total flecainide concentration rose from 1512 ng/ml on admission to 2699 ng/ml 9 hours postingestion. However, in both cases, the interpretation of the increase of flecainide concentrations should be cautious as the second sample could have been drawn during the absorption phase of flecainide. In our patient, the increase of serum flecainide concentration could not be ascribed to a prolonged intestinal absorption and the close temporal relationship with IFE administration suggests a sequestration into the intravascular lipid compartment. However, as in other publications, we were not able to determine free flecainide levels as the only pharmacologically active concentrations. We recognize also that the dosage regimen for IFE therapy was excessive in our patient.

Approximately 30% of an oral dose of flecainide is excreted in urine as unchanged drug, and in patients with renal impairment, the total clearance of this drug might fall by approximately 40% [1]. Our patient had ESRD requiring intermittent hemodialysis, and a daily dose of 150 mg flecainide was likely excessive. In addition, genetic factors could also reduce flecainide metabolic clearance. Flecainide is primarily metabolized by hepatic cytochrome P450 CYP2D6 and CYP1A enzymes. The patients with the CYP2D6^∗^1/^∗^1 (two functioning alleles) genotype are considered as extensive metabolizers. Our patient was genotyped CYP2D6^∗^1/^∗^4 (^∗^4 as an inactive allele) and should be classified as an intermediate metabolizer. Impaired CYP2D6 activity reduces flecainide clearance by 21% in intermediate metabolizers [4]. The restoration of hemodynamic conditions by ECLS, and particularly of liver blood flow, was likely associated with a relative preservation of hepatic clearance.

Due to its pharmacological properties, flecainide is not effectively removable by different extrarenal epuration techniques (hemodialysis, hemofiltration, and hemoperfusion), and this has been verified by isolated case reports [5, 6]. Flecainide overdose had not been previously treated with the hemoperfusion cartridge CytoSorb®. The cartridge is characterized by a biocompatible porous polymer absorber with a total surface of exchange of 45,000 m^2^. It mainly absorbs hydrophobic low and middle molecular substances in a concentration-dependent manner. In vitro data suggest that among cardiovascular drugs, digoxin, amlodipine, and verapamil could be effectively removed, while no data were available for flecainide [7]. The total serum flecainide was well above the therapeutic range at the beginning of hemoadsorption. As expected, the maximal extraction ratio was observed over the first hours. With a mean blood flow of 200 ml/min and a mean extraction ratio of 31%, we can estimate that the clearance of the circuit expressed as plasma clearance was 40.3 ml/min over the first 22 hours. However, we agree that the total plasma clearance of flecainide is represented by the sum of the metabolic (hepatic) clearance and of the clearance related to CVVHDF and CytoSorb®. The exact contribution of hemoadsorption to the clinical improvement could not be easily assessed as other therapies were simultaneously applied. This is particularly true for ECLS that has been applied with success in several cases of refractory (but transient) cardiogenic shock [8–10]. The concurrent or sequential use of both IFE and ECLS in case of drug-related cardiovascular toxicity is still uncommon with a possible negative influence of IFE on the tubing, valves, and circuits [11]. The influence of IFE on the CytoSorb® cartridge is also not known. It is also likely that the efficacy of CytoSorb® on flecainide epuration could decrease with time following cartridge saturation.

The limitations of this single observation have to be acknowledged. First, sodium bicarbonate should have been administered earlier, as soon as flecainide toxicity is suspected, and the total amount of IFE here exceeded the recommended dose. As IFE was not the sole medication used during resuscitation, the apparent clinical improvement could not be ascribed to IFE therapy only. Second, the respective role of each intervention (ECLS, CVVHDF, and hemoadsorption) for the hemodynamic improvement remains difficult to establish, but ECLS was undoubtedly the most saving technique. Third, the effectiveness of the CytoSorb® cartridge for flecainide removal cannot be fully appreciated by the calculations of blood extraction as the amount of drug coated on the cartridge cannot be determined.

## Additional Points

*Learning Objective*. Therapeutic use of flecainide in patients with end-stage renal disease may be associated with serious toxicity. When standard therapy is failing following refractory cardiogenic shock, extracorporeal cardiac life support combined with sequestration of flecainide in a lipid phase followed by hemoadsorption could be helpful in selected cases.
